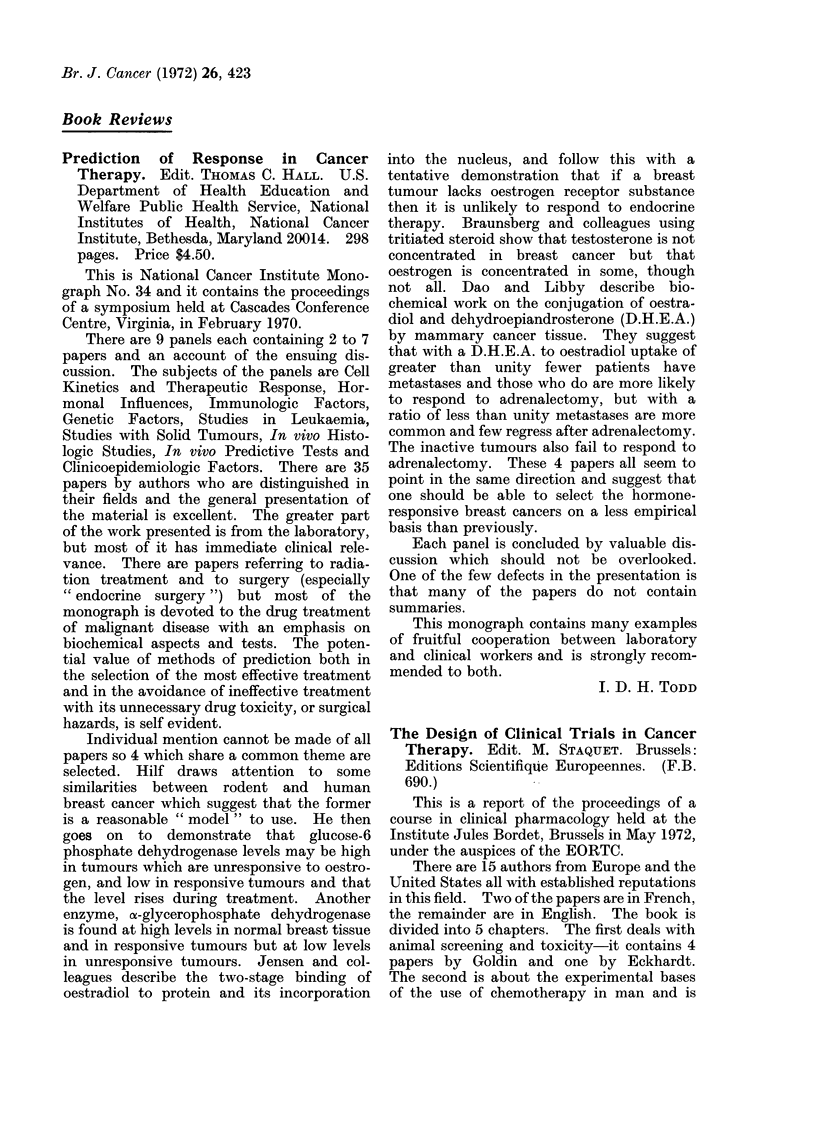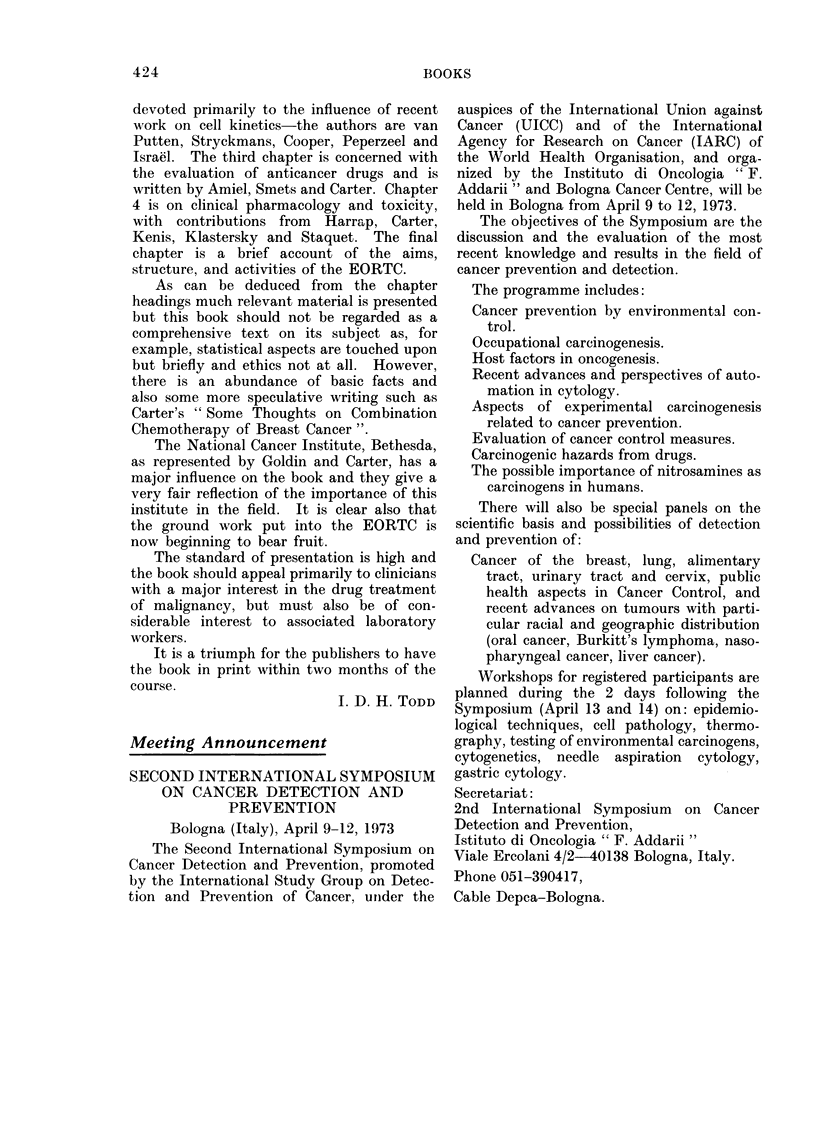# The Design of Clinical Trials in Cancer Therapy

**Published:** 1972-10

**Authors:** I. D. H. Todd


I. D. H. TODD

**The Design of Clinical Trials in Cancer Therapy.** Edit. M. STAQUET. Brussels: Editions Scientifique Europeennes. (F.B. 690.)

This is a report of the proceedings of a course in clinical pharmacology held at the Institute Jules Bordet, Brussels in May 1972, under the auspices of the EORTC.

There are 15 authors from Europe and the United States all with established reputations in this field. Two of the papers are in French, the remainder are in English. The book is divided into 5 chapters. The first deals with animal screening and toxicity—it contains 4 papers by Goldin and one by Eckhardt. The second is about the experimental bases of the use of chemotherapy in man and is devoted primarily to the influence of recent work on cell kinetics—the authors are van Putten, Stryckmans, Cooper, Peperzeel and Israël. The third chapter is concerned with the evaluation of anticancer drugs and is written by Amiel, Smets and Carter. Chapter 4 is on clinical pharmacology and toxicity, with contributions from Harrap, Carter, Kenis, Klastersky and Staquet. The final chapter is a brief account of the aims, structure, and activities of the EORTC.

As can be deduced from the chapter headings much relevant material is presented but this book should not be regarded as a comprehensive text on its subject as, for example, statistical aspects are touched upon but briefly and ethics not at all. However, there is an abundance of basic facts and also some more speculative writing such as Carter's " Some Thoughts on Combination Chemotherapy of Breast Cancer ".

The National Cancer Institute, Bethesda, as represented by Goldin and Carter, has a major influence on the book and they give a very fair reflection of the importance of this institute in the field. It is clear also that the ground work put into the EORTC is now beginning to bear fruit.

The standard of presentation is high and the book should appeal primarily to clinicians with a major interest in the drug treatment of malignancy, but must also be of considerable interest to associated laboratory workers.

It is a triumph for the publishers to have the book in print within two months of the course.

I. D. H. TODD